# Stability analysis and control technology of the sump-surrounding rock under a 1200-m-deep goaf: a case study

**DOI:** 10.1038/s41598-023-36689-x

**Published:** 2023-06-17

**Authors:** Shengrong Xie, Hui Li, Dongdong Chen, Zaisheng Jiang, Junqi Cui, Ruipeng Liu

**Affiliations:** 1 School of Energy and Mining Engineering, China University of Mining and Technology-Beijing, Beijing, 100083 China; 2 Beijing Key Laboratory for Precise Mining of Intergrown Energy and Resources, China University of Mining and Technology-Beijing, Beijing, 100083 China

**Keywords:** Energy science and technology, Engineering

## Abstract

In this study, a sump in the Xingdong coal mine (buried at a depth of over 1200 m) was used to analyze the surrounding rock's stability and control technology. Under the combined influences of various complex conditions, such as the burial depth of over 1200 m, ultra-high ground stress, and location under the goaf, the sump support became extremely difficult, severely restricting the efficient production of the mine. The overall pressure-relief mechanisms and degree of the sump surrounding the rock environment under the goaf were studied, and the rationality of the sump location was verified through numerical simulations and field tests. A more effective support scheme was proposed based on the deformation characteristics and failure mechanisms of the temporary sump-surrounding rock under the supporting conditions. The combined control technology employed the lengthened strong anchor bolts (cables), full-section concrete-filled steel tubular supports, and pouring full-section reinforced concrete and full-section long-hole grouting reinforcement. The field test results showed that after adopting the new support scheme, the sump-surrounding rock tended to be stable after three months. The sump roof subsidence amount, floor heave amount, and convergence of the two sidewalls of the sump were 17.2–19.2 mm, 13.9–16.5 mm, and 23.2–27.9 mm, respectively, thus satisfying the application requirements. This study provides an essential reference for deep-mine roadway support under a complex high-ground-stress environment.

## Introduction

Coal resources remain the primary energy source for developing countries. However, with the increased consumption of shallow coal resources, the maximum coal-mining depth for several countries has exceeded 1000 m^1^, and the deepening of coal mining is gradually becoming a standard practice. The geophysical environments and complex stress fields where deep rock masses are located change the physical and mechanical properties of the rock mass and cause the surrounding rock of the deep roadway to exhibit high ground stresses, large deformations, and time effects^2^. In addition, several soft strata (such as mudstone and sandy mudstone) exist in the deep strata, and the poor load-bearing capacity of the roadway-surrounding rock can severely damage the surrounding rock of the deep roadway. The traditional roadway support methods can no longer satisfy the support requirements for deep roadways^3,4^.

Many scholars have conducted a significant amount of research on surrounding rock control for deep roadways. A stability control theory is proposed for deep rock roadway-surrounding rock based on four basic principles: stress state recovery and improvement, surrounding rock reinforcement, fracture consolidation, and damage repair, and stress peak transfer and load-bearing ring expansion, forming a complete set of technologies for controlling deep roadway-surrounding rock in the Huainan mining area (China)^5^. A principle of minimum deformation support is proposed for deep roadway-surrounding rock control and developed a space–time coupling integrated support technology for deep roadway-surrounding rock stability control^6^. It is believed that improving the stress state and mechanical properties of the roadway-surrounding rock, reasonably selecting the supporting form of the roadway and increasing its supportive resistance, and optimizing the roadway section were effective ways for controlling the deep roadway-surrounding rock^7^. It used a high-strength, high pre-stress, high-elongation anchor bolts (cables) and grouting-combined support and reinforcement technology to significantly reduce the roadway-surrounding rock deformation in a mine with a depth of over 1000 m, and it kept the surrounding rock stable for a long time^8^. Some scholars^9–13^ studied the failure mechanisms of a deep soft rock roadway and proposed various support measures for a deep smooth rock roadway based on the large deformation, overall convergence deformation, and surrounding rock weighting of the deep soft rock roadway. In addition, research has also been conducted on the stability of the surrounding rock of the chamber with a deep drainage system of over 1000 m^14^. Roadway excavation and coal seam mining can lead to rock mass fragmentation and fissure development around a roadway; grouting to fill the internal gaps of the fractured rock mass can effectively improve the load-bearing power of the fractured surrounding rock mass, and give full credence to the total load-bearing capacity of the surrounding rock and the supporting body of the roadway^15–21^. In addition, given the large deformations in deep roadway-surrounding rock, it discussed the supporting mechanisms of a concrete-filled steel tubular support in a deep roadway. It developed a new concrete-filled steel tubular support structure that cooperated with other supporting measures to a certain extent^21^. Accordingly, they could control the large long-term deformations of the deep roadway^22^. Based on their research findings, the above scholars have proposed supporting theories and complementary technologies for controlling deep roadway-surrounding rock.

In contrast to the engineering practices corresponding to the above studies, the sump under the 1200-m-deep goaf in the Xingdong coal mine faces the comprehensive influence of various special geological and engineering conditions. The details are as follows. ① The sump is buried at a depth of over 1200 m in a highly high-ground stress environment, and the surrounding rock is affected by long-term erosion of the water; ② The internal and external sump roadways are long, and the layout of sump roadways is annular. The maximum horizontal principal stress direction at different positions of the sump roadway is not consistent with the axial direction of the roadway, and the sump roadway is affected by a complex stress environment; ③ The water storage function of the sump roadway makes it difficult to renovate and has a long service time; ④ The sump is a roadway with large deformation in soft rock, and conventional support measures are challenging to support. This paper proposes to arrange the sump under the goaf and studies the pressure relief mechanism and degree of the surrounding rock of the sump under the goaf. A combined control technology was proposed for the new sump, including the lengthened strong anchor bolts (cables), full-section concrete-filled steel tubular supports, and pouring full-section reinforced concrete and full-section long-hole grouting reinforcement. By studying the deformation and failure characteristics of the surrounding rock and supporting body of the sump after excavation, an excellent supporting effect was determined to have been achieved in the sump.

## Engineering background

### Geological survey

**Figure 1:**
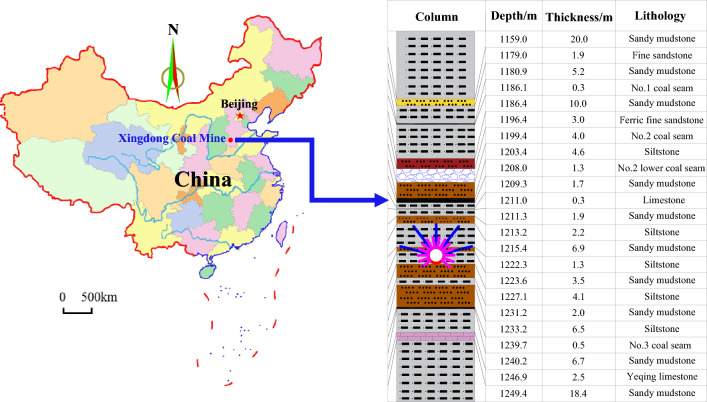
Location of the mine and lithology of the rock strata.

The Xingdong coal mine is situated northeast of Xingtai City, approximately 4.0 km away from the center of Xingtai City. The minefield is about 4.1 km long from north to south and 4.0 km wide from east to west, with an area of approximately 14.5 km^2^ and a ground elevation from + 56.5 to + 58 m. The mine adopts a multi-level development mode around a vertical shaft, with a ground elevation of − 760 m at the No. 1 mining level and a ground elevation of − 980 m at the No. 2 mining level. The two levels are connected through a blind inclined drift. Currently, the No. 2 coal seam is mined at these two levels. The average mining height is 4.0 m, and the average dip angle of the coal seam is 13° in the No. 1 mining district of the No. 2 mining level. The location of the Xingdong coal mine and a geological column chart of the rock strata in the No. 1 mining district of the No. 2 mining level is shown in Fig. 1.

### Engineering survey

**Figure 2:**
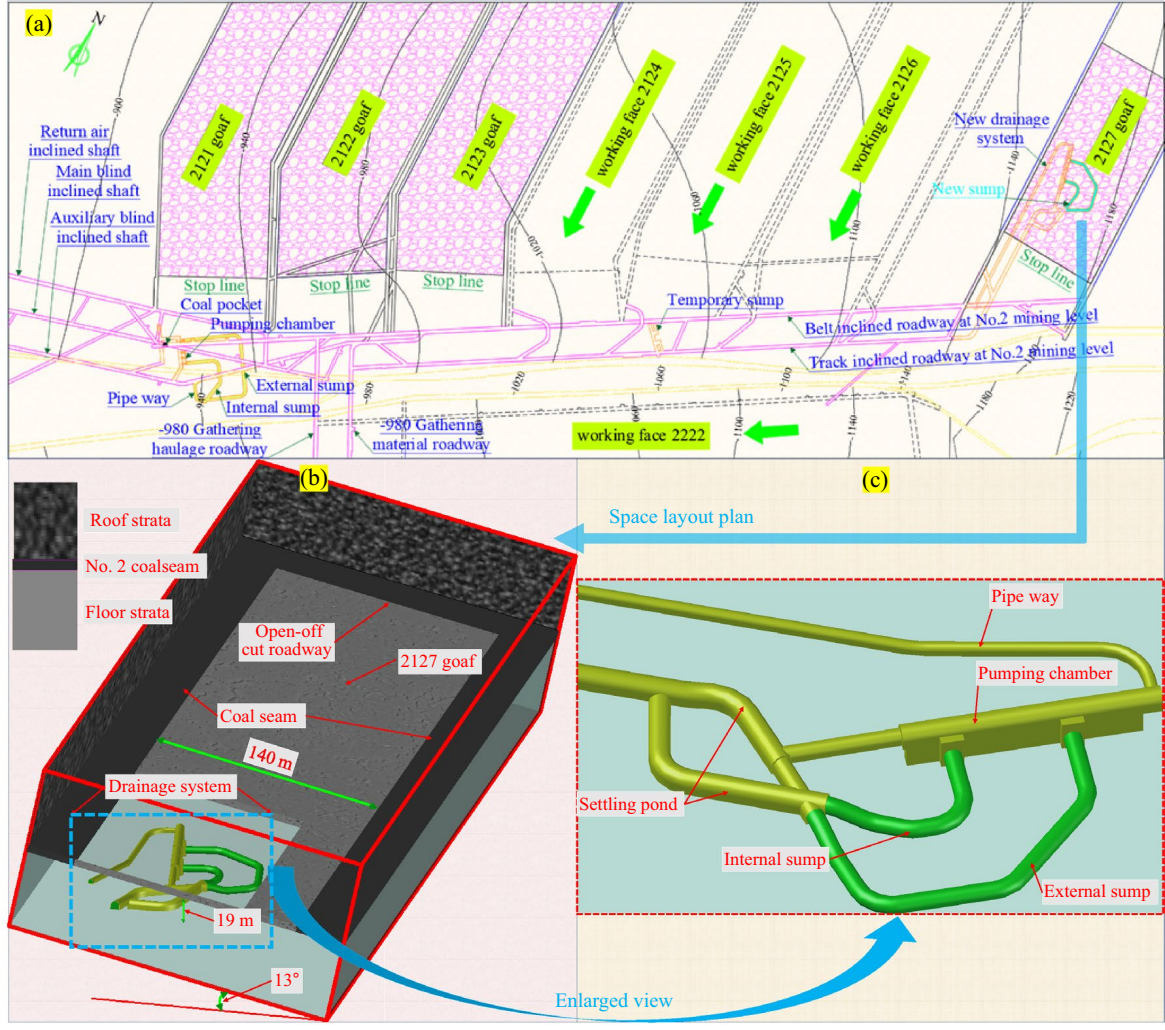
The structure and location of the new drainage system: (**a**) mining plan of 2127 panel. (**b**) Location diagram of d new drainage system. (**c**) Three-dimensional schematic diagram of the new drainage system.

During the excavation and mining at the No. 2 mining level of the Xingdong coal mine, drainage was performed using a temporary sump between the belt inclined roadway and track inclined roadway and near the 2125 district sublevel. The layout of the No. 1 mining district of the No. 2 mining level is illustrated in Fig. 2a. In addition, different extents of the gushing water phenomena have been observed at mined working faces 2123 and 2127; the maximum inflow of water at working face 2127 was 210 m^3^/h. According to the hydrogeological conditions and actual water gushing situations in the Xingdong coal mine, the F_19_ fault (located at the open-off cut roadway side of the No. 1 mining district at the No. 2 mining level) exhibits strong water conductivity. Although the sizeable waterproof coal pillar was retained in the original design, considering the actual situation of the No. 1 mining district and each working face, working faces 2124, 2125, and 2126 encountered severe water-gushing risks. Moreover, the capacity of the original temporary sump was limited and was far from satisfying the drainage demand. Therefore, to ensure safe mining, it is necessary to construct a new drainage system located at deeper positions at working faces 2124, 2125, and 2126 to satisfy the demand.

The field data are presented below ① In the process of extending the main and auxiliary blind inclined shafts to the No. 2 mining level in the Xingdong coal mine, the surrounding rock deformation rate of the shafts gradually increased, and the surrounding rock deformation rate in the middle-lower zone of the shafts was maintained at 2.6 mm/d for a long time. ② The deformation and failure of the surrounding rock at the − 980 m gathering haulage roadway were severe, and the support system was damaged in several places, including the pullout of the anchor cable and the warping of the bearing plate of the anchor cable. Large deformations continued to occur in the surrounding rock of the roadway after two refurbishments. ③ After completing the first excavation of the pumping chamber of the drainage system at the No. 2 mining level (buried at a depth of approximately 1000 m), there was no time to install the equipment. Additionally, a large deformation occurred on the two sidewalls (with a maximum displacement of 1.35 m) and floor (with removal of 1.75 m) of the pumping chamber. To sum up, the ground pressure environment faced by the new drainage system posed significant challenges to its roadway design and support method. Based on the coal seam occurrence, mining layout, hydrogeology, transportation, and other factors, the new drainage system was set in the floor rock strata of the 2127 goaf (as shown in Fig. 2b).

Throughout the drainage system construction roadway (as shown in Fig. 2c), the excavation length of the internal and external sumps accounted for 61% of the total excavation length for the drainage system. After excavating the sump, the surrounding rock underwent hydration swelling, softening, and disintegration (or even marginalization) owing to pressure relief, weathering, and especially long-term water erosion. These led to a reduction in strength and severely affected the self-load-bearing effect of the sump-surrounding rock^23^. Therefore, maintaining the surrounding rock mass of the sump effectively over a long duration was necessary for the drainage system to sustain regular operation.

The buried depth of the sump reached 1200 m, and the virgin rock stress alone could get 30 MPa. Excavation of the sump in such a high-virginity rock stress field could reduce the lateral pressure of the surrounding rock within a certain radius around the sump; simultaneously, the stress concentration caused by the stress redistribution after the excavation could increase the circumferential stress of the sump by 2–3 times. Owing to the prominent contradiction between the high stresses caused by the decrease of the lateral pressure, the increase of the circumferential pressure in the sump-surrounding rock, and the low strength of the surrounding rock mass, the surrounding rock mass would inevitably deteriorate rapidly. This would cause the surrounding rock mass to undergo many instability processes and damage, e.g., damage dilatancy-shear slip failure-fragmentation and expansion deformation. Simultaneously, the stress exceeding the strength of the surrounding rock would transfer to the deep, affecting the stability of the deep surrounding rock^5^. According to the factors affecting the stability of the roadway-surrounding rock and their laws, combined with the deep production geological conditions of the Xingdong coal mine, the strength of the sump-surrounding rock can be controlled from the following two aspects^6^: ① improving the stress state and mechanical properties of the roadway-surrounding rock; ②selecting the appropriate supporting method, and improving its supportive resistance.

### Analysis of pressure relief in the surrounding rock environment of the sump

The new sump was arranged in the floor rock strata of the 2127 goaf, i.e. in a pressure-relief area. Accordingly, it could effectively improve the stress environments of the roadway space, surrounding rock fracture damage area, and broader rheological influence area^24^, relieve severe large deformations of the roadway-surrounding rock under high ground stress, and reduce the difficulties in supporting the roadway-surrounding rock.

### Numerical model

**Figure 3:**
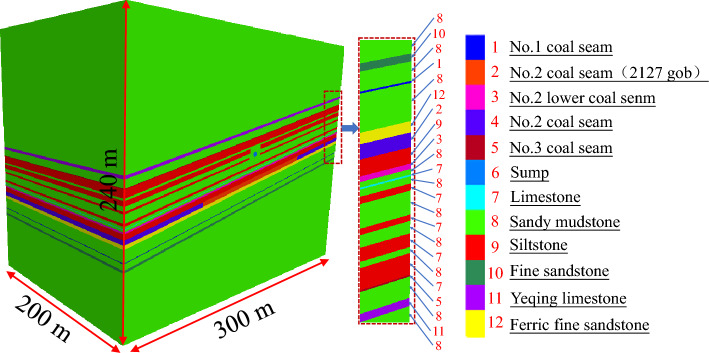
Numerical simulation model.

Based on the simplified calculation and the geological conditions of the working face 2127 in the No. 1 mining district of the No. 2 mining level, the FLAC^3D^ numerical simulation software was used to study the stress distribution laws of the floor strata after the mining of working face 2127; this was done to compare the stress distribution of the sump-surrounding rock before and after the mining and to analyze the degree of pressure relief for the rock strata at the sump position. The model size was 300 × 200 × 240 m; the dip angle of the coal seam and other rock strata was 13°; the length of the working face was 140 m; and the mining height was 4.0 m (The cross-sectional shape of the sump in the model is circular). The transverse boundary of the model was fixed in the horizontal direction, and the bottom boundary was fixed in the vertical direction. According to the in-situ stress measurement results, a vertical stress of 27.5 MPa was applied to the upper boundary of the model to simulate the weight of the overburden. The lateral pressure coefficient of the model is 1.2. The model is illustrated in Fig. 3.

### Selection of mechanical parameters and constitutive model of rock stratum

#### Mechanical properties of rock stratum and strain-softening model

The Mohr–Coulomb model was used for the rock strata, the strain-softening model for the yield coal pillar, and the double-yield model for the goaf.

There are many irregular joints and cracks in the coal and rock mass. The parameters measured through experiments are usually higher than those of the rock mass in the stope. A reliable estimation of the rock’s mechanical parameters is essential for obtaining accurate results in the numerical simulation. It studied and analyzed a variety of numerical simulation examples for rock structures^25^. It was found that the numerical model stiffness value was, on average, 0.469 of the laboratory stiffness value. On average, the model uniaxial compressive strength value was 0.284 of the laboratory strength value. By estimating the mechanical parameters of the coal and rock masses used in the numerical model^26,27^, it was determined that the elastic modulus, cohesion, and tensile strength values corresponded to 0.2 of the laboratory testing result values and that the Poisson's ratio was 1.2 of the laboratory testing result value.

The coal body around the goaf reached peak stress due to mining the No. 2 coal seam. The uniaxial compressive strength of the coal body decreased rapidly, and the coal body was destroyed; this process is known as “strain‐softening.” The strain-softening model assumes that when the rock mass is damaged, the mechanical parameters of the rock mass change with the strain and then remain unchanged after reaching a residual stage^28^. The residual elastic modulus, cohesive force, and internal friction angle needed for the strain-softening model for coal were obtained from experiments^29^. The mechanical parameters of each stratum are shown in Table 1.

**Table 1:** Mechanical parameters of rock strata.

Rock strata	${\text{K}}$/GPa	${\text{G}}$/GPa	${\text{C}}_{\text{m}}$/MPa	${\sigma }_{\text{tm}}$/MPa	${\varphi }_{\text{m}}$/(°)	${\text{D}}$/(kg·m^−3^)
Sandy mudstone	4.87	2.58	1.20	1.00	30	2.40
Fine sandstone	5.82	3.14	1.50	1.20	32	2.55
Coal seam	3.75	1.87	0.90	0.70	27	1.40
Siltstone	5.32	2.84	1.30	1.10	31	2.65
Yeqing limestone	4.42	2.40	1.10	1.10	30	2.60
Limestone	4.70	3.10	3.20	1.20	32	2.10
Ferric fine sandstone	6.87	4.38	2.40	1.40	29	2.40

${\text{K}}$ is the bulk modulus; ${\text{G}}$ is the shear modulus; ${\text{C}}_{\text{m}}$ is the cohesion; ${\sigma }_{\text{tm}}$ is the tensile strength; ${\varphi }_{\text{m}}$ is the friction angle; and ${\text{D}}$ is the density.

### Double-yield model

After the coal seam was mined, the roof of the goaf collapsed. With the continuous advance of the working face, the collapsed gangue in the goaf was gradually compacted, and its modulus was significantly increased^30^. The collapsed gangue in the goaf bore part of the vertical load, which not only effectively relieved the stress concentration of the coal around the goaf^31^ but also affected the stress distribution law of the floor strata, thereby affecting the numerical simulation results. Therefore, the goaf model required consideration. In this study, the double-yield model was used to describe the coal gangue collapse and compaction process in the goaf^32–34^, and then a good stress distribution law was obtained for the floor strata in the goaf.

According to existing research^35^, the double-yield model requires cap pressure and material properties. Accordingly, the Salamon equation^36^ can be used to estimate the cap pressure parameters as follows:

1 $$\sigma = \frac{{\mathop E\nolimits_{0} \varepsilon }}{{1 - \frac{\varepsilon }{{\mathop \varepsilon \nolimits_{\max } }}}}.$$

In the formula, $\sigma$ is the stress applied to the goaf materials; ${E}_{0}$ is the initial tangential modulus of the goaf materials; $\varepsilon$ is the volumetric strain under the applied pressure; and ${\varepsilon }_{max}$ is the maximum volumetric strain of the goaf materials.

${E}_{0}$ and ${\varepsilon }_{max}$ can be determined by the in-situ stress and bulking factor^30^, as follows:

2 $$\mathop E\nolimits_{0} = \frac{{10.39\mathop \sigma \nolimits_{c}^{1.042} }}{{\mathop b\nolimits^{7.7} }},$$

3 $$\mathop \varepsilon \nolimits_{\max } = \frac{b - 1}{b}.$$

In the formula, ${\sigma }_{c}$ is the compressive strength of the rock pieces; and $b$ is the bulking factor. The value of $b$ can be determined as follows:

4 $$b = \frac{{\mathop h\nolimits_{m} + \mathop h\nolimits_{cz} }}{{\mathop h\nolimits_{cz} }}.$$

In the formula, ${h}_{m}$ is the mining height; and ${h}_{cz}$ is the height of the caved zone.

The height of the caved zone can be determined based on the following empirical equation^37^:

5 $$\mathop h\nolimits_{cz} = \frac{{100 \cdot \mathop h\nolimits_{m} }}{{4.7 \cdot \mathop h\nolimits_{m} + 19}} \pm 2.2.$$

Substitute *h*_m_ into formula (5) to obtain a maximum height of 12.78 m for the caved zone, and the uniaxial compressive strength of the caved rock in the goaf (obtained through the laboratory test) was 10.73 MPa. By substituting the above data into the above Eqs. (1), (2), (3) and (4), the goaf materials’ maximum volumetric strain and initial modulus were estimated as 0.23 and 16.26 MPa, respectively. The cap pressure used in the double-yield model is listed in Table 2.

**Table 2:** Cap pressure used in the double-yield model.

Strain	Stress/MPa	Strain	Stress/MPa
0.01	0.17	0.12	4.08
0.02	0.36	0.13	4.86
0.03	0.56	0.14	5.82
0.04	0.79	0.15	7.01
0.05	1.04	0.16	8.55
0.06	1.32	0.17	10.6
0.07	1.64	0.18	13.46
0.08	1.99	0.19	17.76
0.09	2.4	0.20	24.92
0.10	2.88	0.21	39.27
0.11	3.41		

**Figure 4:**
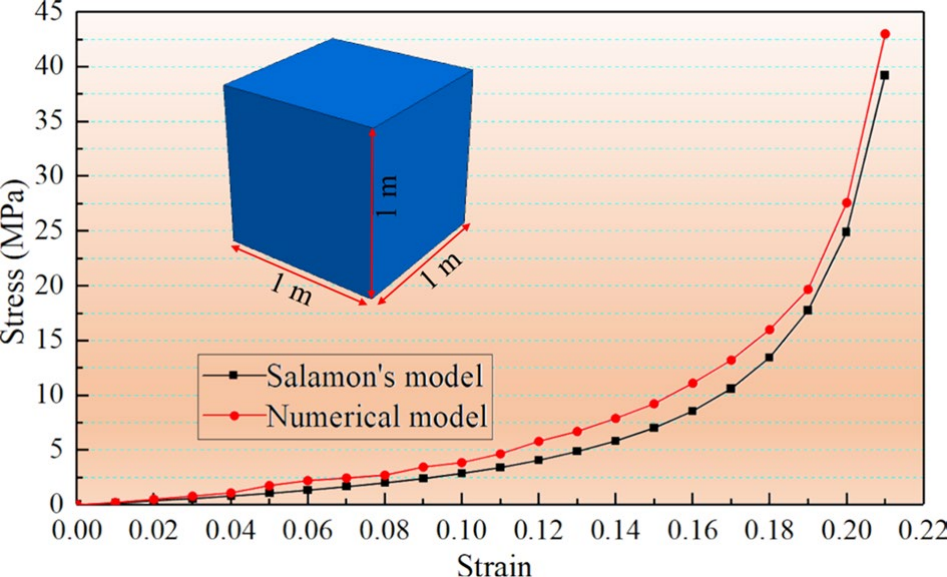
Comparison of the stress–strain curve between the numerical model and Salamon's model.

The parameters used in the double-yield model were obtained using a trial-and-error method. In addition, a cube model with dimensions of 1 × 1 × 1 m was generated in the numerical simulation software. A constant velocity was applied at the top of the model to simulate the vertical loading; the horizontal displacement of the four vertical planes of the model and the vertical displacement of the bottom boundary were fixed. To fit the stress–strain curves of Salamon's model, the parameters used in the double-yield model were repeatedly adjusted (as shown in Fig. 4). Table 3 presents the calibrated properties for the goaf materials.

**Table 3:** Material parameters used in the double‐yield model.

Parameter	${\text{D}}$/(kg·m^−3^)	${\text{K}}$/GPa	${\text{G}}$/GPa	${\varphi }_{\text{m}}$/(°)	Dilation/(°)
Value	1100	1.21	0.53	20	6

## Results and analysis

**Figure 5:**
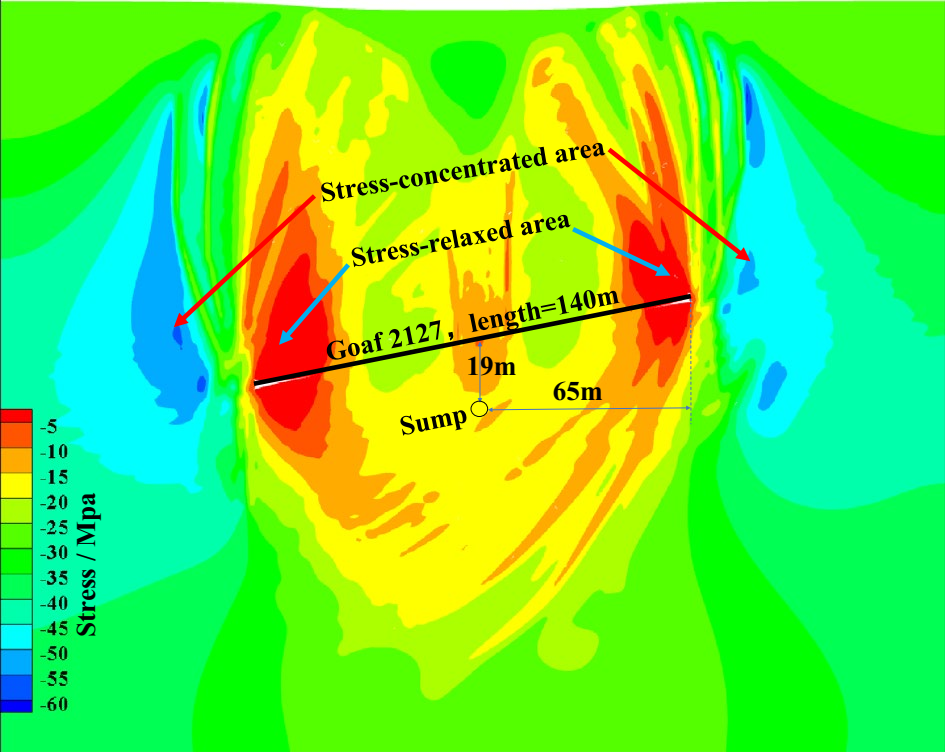
Vertical stress contour after working face 2127 mining.

It was known that before working face 2127 was mined, the floor rock strata of the pre-mining working face were in the pristine rock stress state, and the surrounding rock pressure of the sump exceeded 30 MPa. After working face 2127 was mined, a stress-concentrated area was formed in the inclination direction of the working face. In addition, a stress-release area was created in the two end areas of the goaf. A pressure-relief area was formed in the goaf's middle and floor rock strata (as shown in Fig. 5). The stress range of the pressure-relief area in the floor rock strata of the goaf was 10–20 MPa. The deformation and failure of the sump-surrounding rock caused by the high stress could be effectively reduced by arranging the sump in this area. Compared with the virgin rock stress of approximately 30 MPa, the low-stress area in the goaf floor effectively improved the high-stress surrounding rock environment of the sump (Providing a good stress space for the support structure of the sump), alleviated the rheological effects caused by the deep high stress, and became a powerful barrier for resisting the continuous and large deformations in the rock area surrounding the sump. The surrounding rock of the pressure-relief area in the goaf floor underwent mining-induced effects such as damage dilatancy, shear slip failure, fragmentation, and expansion deformation; thus, the area became a fracture-development area. Subsequently, as the caving rock mass in the goaf compacted the coal and rock mass of the floor, the fissures closed to a certain extent, and the stress gradually returned to the virgin rock stress state^38^.

Nevertheless, the creep characteristics of the rock mass in this area were substantially weakened owing to the pressure relief and expansion of the rock strata, and the stress values in this area were high only in the local range. After excavating the sump, no continuous damage was caused by the high deviatoric stress in the adjacent area. Moreover, the caving zone in the upper goaf served as a cushion layer. It could weaken or eliminate the continuous and large deformations of the surrounding rock caused by the rheological characteristics of the deep rock mass.

**Figure 6:**
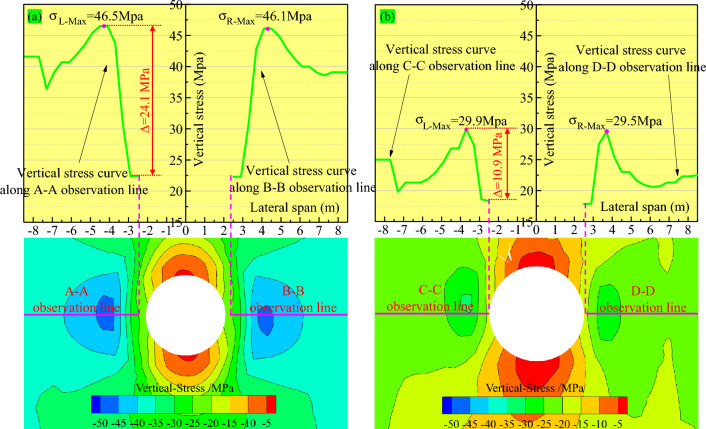
Stress contour of the sump. (**a**) Stress contour before mining. (**b**) Stress contour after mining.

A circular roadway was excavated in the floor rock strata of working face 2127 under mining and non-mining conditions to simulate the sump's excavation and analyze the stress distribution around it. The stress contour lines of the shallow surrounding rock of the sump without pressure relief were relatively dense and had an extensive range. The stress difference value was approximately 25 MPa in the field of 2.2 m, and the maximum concentrated stress was 46.5 MPa (in Fig. 6a). The stress gradient of the shallow surrounding rock of the sump after pressure relief was relatively small. The stress difference between the two sides of the sump decreased to approximately 15 MPa, and the maximum concentrated stress was 29.9 MPa (in Fig. 6b). The overall pressure relief amplitude was 35.7%. In general, the pressure relief of the sump-surrounding rock significantly reduced the stress concentration degree of the surrounding rock. Moreover, the pressure relief on the sump-surrounding rock was very significant to the support of the sump-surrounding rock in later periods and to the realization of the long-term stability of the sump.

### Deformation characteristics and failure mechanisms of surrounding rock of the 1050-m-deep temporary sump

Through clarifying the support mode and failure characteristics of the 1050-m-deep temporary sump, the mechanism of the sump surrounding rock instability is described, and guiding suggestions are put forward for surrounding rock support of the deeper sump.

### Support patterns and parameters of the 1050-m-deep temporary sump

**Figure 7:**
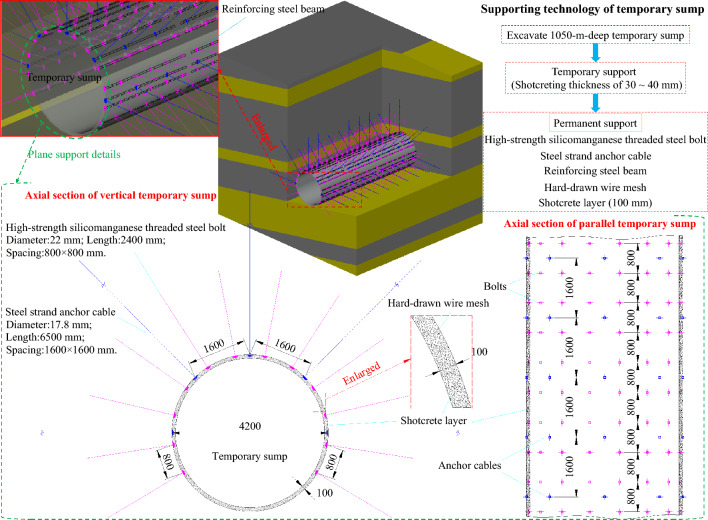
Support scheme of the temporary sump (unit: mm).

As mentioned above, the temporary sump between the belt inclined roadway and track inclined roadway is located at the No. 2 mining level and near the 2125 district sublevel, which a buried depth is about 1050 m. Shotcreting was initially used for temporary support after excavating the temporary sump, with an initial shotcreting thickness of 30–40 mm. Then, bolting and shotcreting with a wire mesh were used for permanent support. High-strength silicomanganese threaded steel bolts (specified as Ø22 × 2400 mm) were used, with spacing and row spacing of 800 × 800 mm; simultaneously, reinforcing steel ladder beams (specified as Ø14 × 4200 mm) and vaulted bearing plates with Ø6 mm hard-drawn wire meshes were used for reinforcement, to protect the surrounding rock surface. Steel strand anchor cables (specified as Ø17.8 × 6500 mm) were used to strengthen the support, with spacing and row spacing of 1600 × 1600 mm. There were five anchor cables in each row, and they were used together with steel bearing plates and wooden backing plates. The strength grade of the concrete used for shotcreting was C20 (The compressive strength is 20 MPa), and the thickness of the shotcreting concrete was 100 mm. The support scheme of the temporary sump is shown in Fig. 7.

### Failure characteristics of the surrounding rock of the temporary sump

Soon after the completion of the support scheme of the temporary sump, the surrounding rock of the temporary sump produced severe deformations under the actions of high-ground stress. The roadway roof subsided over a large area, the two sides converged violently, and the floor heave was severe. The support system of the temporary sump was seriously invalid; the top shotcreting layer of the roadway sidewalls cracked and peeled off, and the reinforcing steel mesh was torn, with an evident mesh pocket. The anchor cables were severely damaged, and the bearing plates were warped in several sump areas.

**Figure 8:**
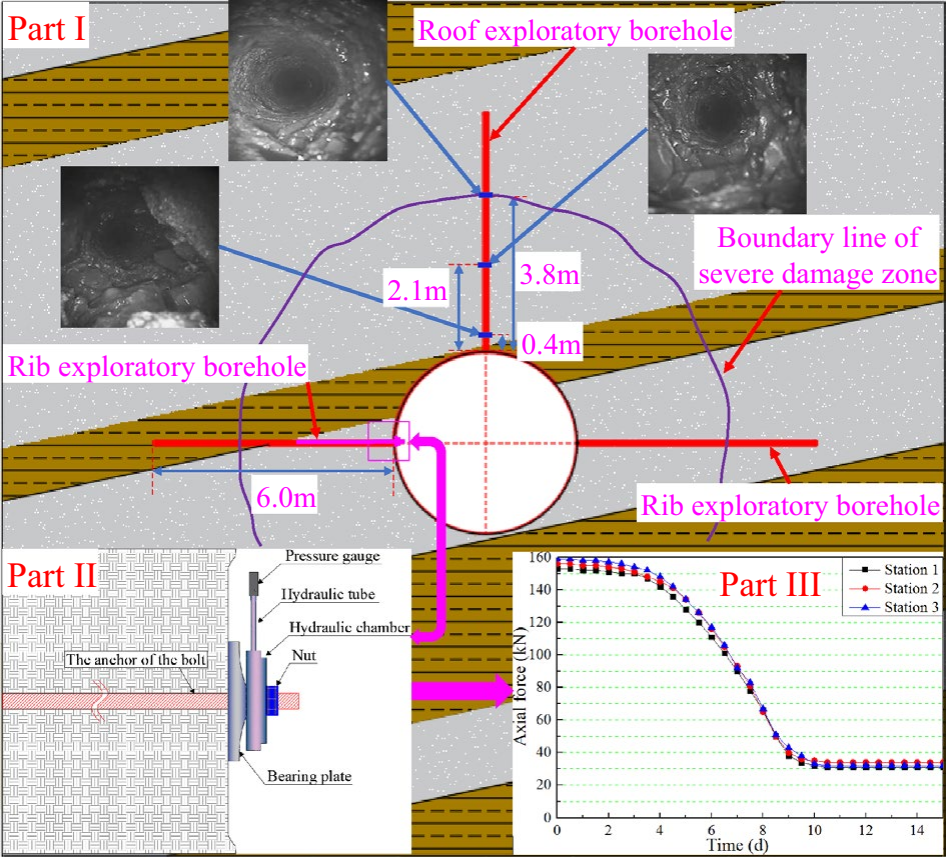
Monitoring of surrounding rock and support damage.

To better obtain the fissure developments in the sump-surrounding rock, a YSZ(B) panoramic drill hole camera system was used to measure the fracture zone development of the roof and two sides of the temporary sump. The depth of each exploration drill hole was 6.0 m, and the diameter of the drill hole was 28 mm. The layout of the exploration drill holes and the detection results for the surrounding rock fissure development are illustrated in Fig. 8-Part I. It was found that the surrounding rock fracture ranges of the roof and two sides of the sump were almost identical; the rock mass was severely damaged at a depth of 0–2.1 m in the drill hole, and the fissure density gradually decreased from the depth of 2.1 m to 3.8 m. At depths of 3.8 m or more, there were only a few fractures in the rock mass.

In addition, as illustrated in Fig. 8-Part II, pressure gauges were used to regularly test the support resistance of the bolt-bearing plate to the surrounding rock through the arrangement of measuring stations (three stations in total, with each measuring station equipped with five pressure gauges). The detection results showed that the anchor force of the bolts gradually decreased with time (the average change values from each measuring station are shown in Fig. 8-part III). The final results showed that the bolts failed to effectively anchor the separated rock mass and could not provide a surrounding rock-strengthening effect.

### Analysis of instability mechanisms of the temporary sump and support suggestions for the 1200-m-deep sump

Based on the field investigation and monitoring analysis, the main reasons for the instability of the temporary sump are summarized as follows.

1. Broken surrounding rock mass: The stress was redistributed after excavating the soft rock roadway with high-ground stress. The roof and two sides of the roadway all experienced severe shear slip failure. Finally, they entered a yielding state under the double action of highly concentrated stress and soft rock characteristics. This resulted in the loss of the self-load-bearing capacity of the rock mass.
2. Creep characteristics of the surrounding rock: The self-load-bearing capacity of the surrounding rock was weak after excavating the soft rock roadway, and an evident creep phenomenon would occur in the surrounding rock once the roadway was excavated. The surrounding rock of the temporary sump would also maintain a continuous large deformation for a long time, allowing the damage scope of the temporary sump to continue to expand.
3. Hydrological effect: Due to the long-term storage and drainage of the temporary sump, the free surface of the temporary sump was in contact with mine water for a long time, severely weakening its strength. Furthermore, the development of fissures in the shallow surrounding rock resulted in strong water conductivity, further weakening the strength of the deep surrounding rock.
4. Support forms and parameters: Reasonable support forms and parameters could effectively control the deformation of the sump-surrounding rock. However, if the support design was unreasonable or the support strength was insufficient, the deformation of the surrounding rock would fail to be effectively controlled; moreover, the support would also be seriously damaged.

In summary, the high stress, soft rock, hydraulic failure, and other factors caused the strength of the surrounding rock to plummet, such that the load that the surrounding rock itself should have borne was transferred to the support. This increased the deformation pressure of the support, ultimately causing the anchor cables to be pulled off and the shotcreting layer to crack and peel off.

The strength, stiffness, stability, and other aspects of traditional bolting and shotcreting with wire meshes do not match the surrounding rock pressure and soft rock characteristics of the temporary sump, and the supporting effect cannot meet the requirements. The support difficulty is more severe for the permanent sump with an enormous buried depth and more complex production geological conditions. Combined with the support parameters of the 1050-m-deep temporary sump and the instability mechanism of surrounding rock, we put forward the following suggestions for the support system of the permanent sump under the 1200-m-deep goaf. ① Bolts (cables) with larger parameter values (such as more considerable length and pre-tension force) should be selected to increase the support strength and anchor range of the bolts (cables) based on the bolts (cables) support of the temporary sump. ② Grouting reinforcement should be used to fill in the fissures in the surrounding rock. This will cause the broken surrounding rock to form a relatively stable mechanical structure, enhancing the surrounding rock's strength and stability. Then, the bolts (cables) can be anchored into the dense and stable rock mass via grouting reinforcement in the surrounding rock and can form a specific coordination with the surrounding rock. This arrangement gives full play to the load-bearing characteristics of the surrounding rock. ③ Considering the broken rock mass and hydrological effect, the concrete pouring should be conducted on the free surface of the surrounding rock of the sump to prevent direct contact between the water, air, and surface of the surrounding rock.

### Supporting system for the surrounding rock of the sump and its application

#### Supporting principles

The combined control technology was proposed based on analyzing the failure characteristics and mechanisms of the temporary sump. It comprised the lengthened strong anchor bolts (cables), full-section concrete-filled steel tubular support, pouring full-section reinforced concrete, and full-section long-hole grouting reinforcement. This approach was combined with data regarding the development status of the industry and advancements in support materials.

The outstanding features of this technology are as follows. First, the lengthened strong anchor bolts (cables) could not only improve the stress state of the surrounding rock but could also inhibit the discontinuous deformation of the shallow surrounding rock to the greatest extent and could improve the strength of the surrounding rock^39^. The full-section concrete-filled steel tubular support could provide a large supporting reacting force to resist the enormous deformation pressure caused by releasing the plastic energy of the surrounding rock. The pouring of the full-section reinforced concrete and full-section long-hole grouting reinforcement could enhance the overall performance of the shallow surrounding rock and cause the shallow surrounding rock and support system to form a whole to ensure the long-term stability of the roadway. The reasonable matching of the four supporting methods regarding time, space, and technological processes could effectively control the large deformations of surrounding rock in a deep roadway.

#### Supporting technology

**Figure 9:**
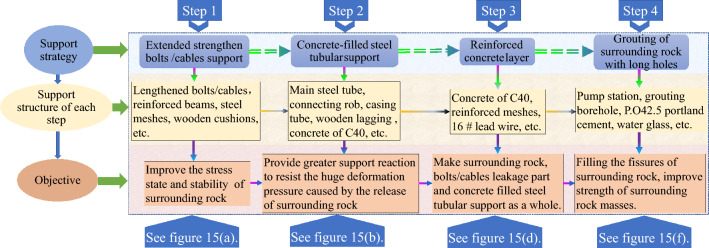
Detailed steps for performing control strategies.

The combined control technology described in this section was used to support the sump, and the detailed technological process is shown in Fig. 9.

**Figure 10:**
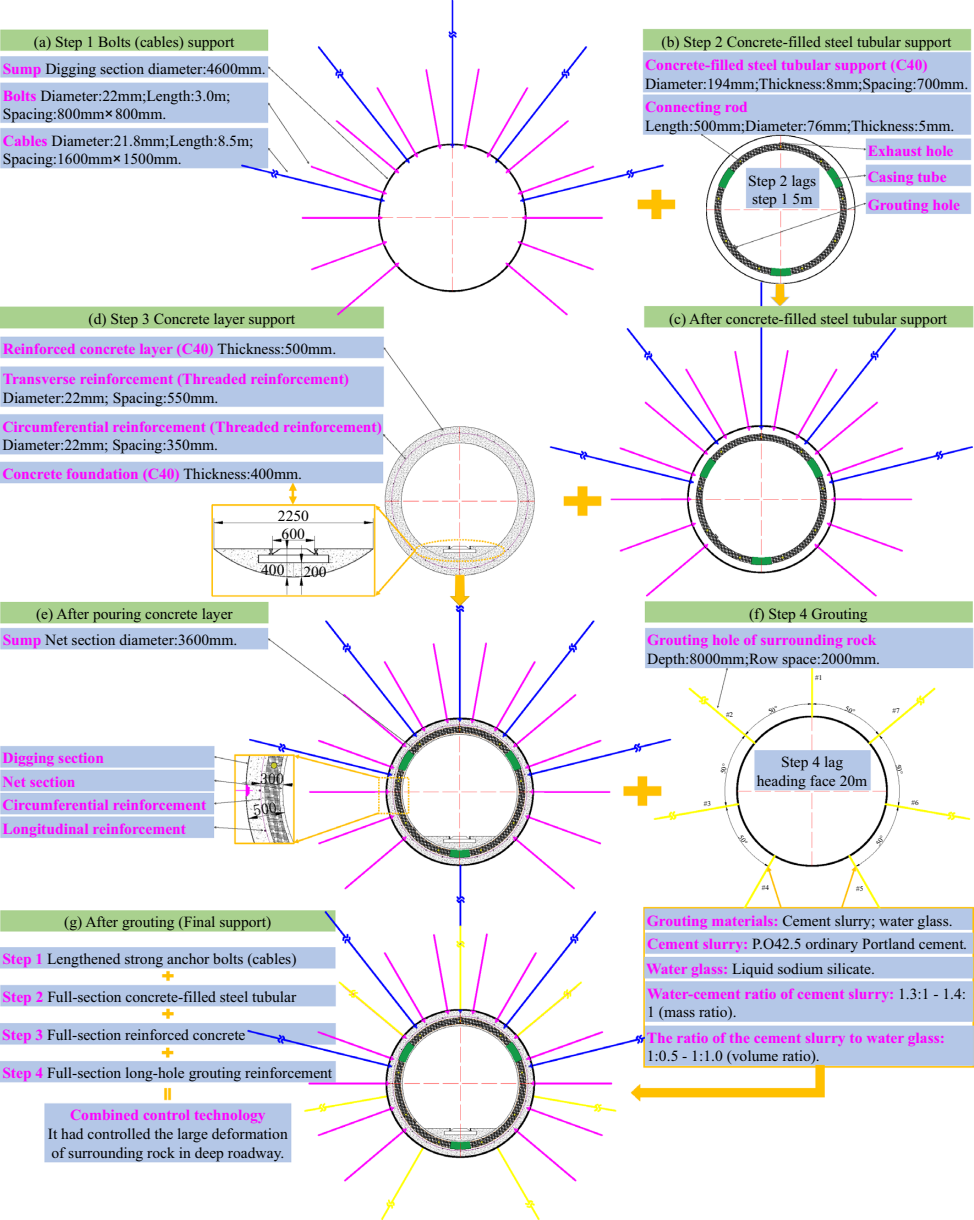
Detailed support scheme in the sump.

Step 1: Initially, the lengthened strong anchor bolts (cables) were used for support. Ultra-strong threaded steel bolts (specified as Ø22 × 3000 mm) were used, with spacing and row spacing of 800 × 800 mm. The anchoring in each hole was implemented using a roll of S2360 capsule resin and a roll of Z2360 capsule resin. Then, the Ø14 mm reinforcing steel ladder beams were combined with the vaulted bearing plates and Ø6 mm hard-drawn wire mesh. The reinforcing steel ladder beams needed to overlap, and the overlapped parts required compression with anchor bolts. Steel strand anchor cables (specified as Ø21.8 × 8500 mm) were used to strengthen the support, with spacing and row spacing of 1600 × 1500 mm. The anchoring in each hole was implemented using a roll of S2360 capsule resin and two rolls of Z2360 capsule resin. Then, 2.6-m-long 14^#^ steel channels were used to link the anchor cables and worked in coordination with the steel bearing plates and wooden backing plates. The pre-tension forces of the bolts and anchor cables were 90 kN and 250 kN, respectively. The pre-tension force of the bolts (cables) could effectively improve the integrity of the shallow surrounding rock and give full play to the self-load-bearing capacity of the deep stable rock mass. After this step’s implementation, the sump's cross-section support diagram is shown in Fig. 10a.

Step 2: The full-section concrete-filled steel tubular support was used for secondary support, lagging approximately 5 m behind the bolts (cables) support after the primary support was completed. The support (as shown in Fig. 10b) was composed of three sections: the top arch section, the bottom section on the left side of the roadway, and the bottom section on the right side. The main steel pipe of the support was a 20^#^ seamless steel pipe (specified as Ø = 194 × 8 mm) with a unit length mass of 36.7 kg/m; each section of the steel pipe was connected by casing pipe, and the casing pipe was made of a seamless steel pipe (specified as Ø = 219 × 8 mm) with a unit length mass of 41.6 kg/m. The spacing between the supports was 700 mm, and to prevent the supports from toppling owing to the instability of the pressure bars, nine ejector pins were used to connect the supports. The length of each ejector pin was 500 mm, and the ejector pin was made of a short concrete bar (specified as Ø = 76 × 5 mm). After the supports were installed, the wire mesh was laid on the sidewalls and roof of the sump, and the gaps between the support and sump were filled with the back plates and woven bags filled with ballast (ideally, the compact filling was ensured). In addition, after installing 5–10 supports, the supports were grouted to increase the grouting efficiency. The strength grade of the concrete poured into the steel tubes was C40 (The compressive strength is 40 MPa). Based on the use of cement, sand, and gravel as the essential ingredients, a water-reducing agent was added to meet the pumping requirements; an expansion agent was added to prevent the concrete from peeling off from the steel tubes owing to the dry shrinkage of the concrete; and steel fiber was added to increase the toughness and deformation resistance of the concrete. The support diagram of the sump after being supported by the full-section concrete-filled steel tubular support is shown in Fig. 10c.

The maximum support resistance $p$ of the concrete-filled steel tubular support is calculated as follows^40^:

6 $$p = \frac{{\mathop \sigma \nolimits_{0} \mathop N\nolimits_{0} }}{{D\mathop R\nolimits_{S} }}.$$

In the above, ${\varphi }_{0}$ is the reduction coefficient, considering the influencing factors such as the cross-sectional shape and cross-sectional area of the support (in this example, 0.8); ${N}_{0}$ is the maximum bearing capacity of the concrete-filled steel tubular short column under axial compression (2518.4 kN for the model of Ø 194 mm × 8 mm); *D* is the spacing of the support (0.7 m); and ${\text{R}}_{\text{S}}$ is the radius of the support (1.85 m). The maximum support resistance *P* of the concrete-filled steel tubular short column was 1.56 MPa.

Step 3: After the installation of the concrete-filled steel tubular support was completed, a 500 mm-thick C40 concrete layer was poured on the surface of the roadway surrounding rock along the section of the roadway (as shown in Fig. 10d). The purpose was to tightly connect the surrounding rock, exposed parts of the bolts (cables), and concrete-filled steel tubular support to form a whole, and to simultaneously provide an excellent closed environment for subsequent grouting in the surrounding rock. In addition, the tensile capacity of the concrete layer was enhanced by adding a single layer of reinforcing steel mesh before pouring the concrete layer; the reinforcing steel mesh was composed of circumferential reinforcement and transverse reinforcement and was made of Ø22 mm threaded reinforcement. The reinforcing steel mesh was 300 mm away from the outer wall of the concrete layer. The circumferential reinforcement spacing was 350 mm, and the transverse reinforcement spacing was 550 mm. The intersection of the two types of reinforcement was bound using 16# galvanized steel wire. The reinforced concrete layer changed the inherent properties of the shallow surrounding rock of the roadway. The shallow surrounding rock of the sump roadway formed by the reinforced concrete layer has a more vital ability to resist high deviatoric stress fields and damage than the original sump roadway surrounding rock. Thus, the reinforced concrete layer directly and rapidly improved the stress in the shallow surrounding rock, significantly reduced the deviatoric stress generated during the formation of the secondary stress field, and improved the stress state of the surrounding rock. In addition, a 400 mm-thick concrete foundation was built to facilitate the installation of tracks for regular silt removal from the sump. The support diagram of the sump after being supported by the pouring support is shown in Fig. 10e.

Step 4: The grouting reinforcement of the surrounding rock began 20 m behind the heading face. Seven grouting holes were drilled along the section of the roadway (as shown in Fig. 10f), each with a depth of 8 m. The No. 1 drill hole was arranged perpendicular to the roof of the sump; the No. 2, No. 3, and No. 4 drill holes were arranged such that they were rotated counterclockwise by 50° along the No. 1, No. 2, and No. 3 drill holes in turn. The No. 5, No. 6, and No. 7 drill holes were symmetrically arranged with the No. 4, No. 3, and No. 2 drill holes along the central axis of the sump, with a row spacing of 2000 mm. To control the setting time of the grouting materials, a double slurry containing cement slurry and water glass was selected for the grouting filling. The water-cement ratio of the cement slurry was 1.3:1 to 1.4: 1 (mass ratio), and the cement slurry to water glass was 1:0.5 to 1:1.0 (volume ratio). The cement used was P.O42.5 ordinary Portland cement, and the Baume degree of the water glass (liquid sodium silicate) was 35°Bé. The grouting pressure was 4.5 MPa. The effective diffusion radius was not less than 2.8 m by adjusting the permeability of the slurry and final grouting pressure during construction. The broken zone of the surrounding rock formed a whole, and the self-load-bearing capacity of the surrounding rock was improved by filling the fissures and separation zone in the sump-surrounding rock with the double slurry. The support diagram of the sump after being supported by the grouting support is shown in Fig. 10g.

**Figure 11:**
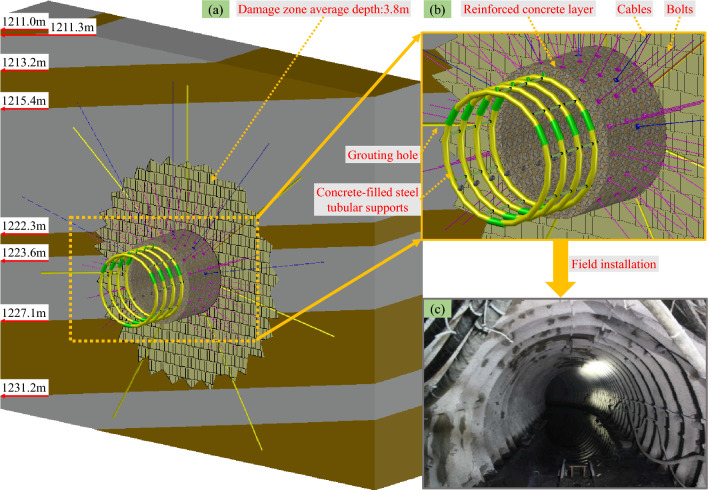
Supporting effect diagram of the sump after multiple support.

A spatial cross-sectional view diagram of the sump after adding the lengthened strong anchor bolts (cables) and full-section concrete-filled steel tubular support and pouring the full-section reinforced concrete and full-section long-hole grouting reinforcement to control the sump-surrounding rock is illustrated in Fig. 11b. A comprehensive diagram of the broken zone range of the surrounding rock after the completion of each support is shown in Fig. 11a. The field construction and installation is illustrated in Fig. 11c.

### Engineering practice

**Figure 12:**
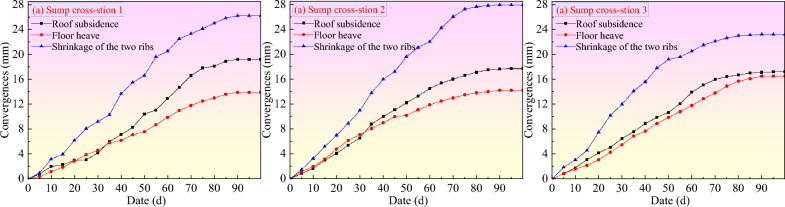
Displacement curve of the sump after multiple support.

To effectively grasp the supporting effect on the sump, three cross-sections of the sump were selected for deformation observation after the completion of the support. The roof subsidence amount, floor heave amount, and convergence between the two sides of the roadway were mainly observed. The measured data are shown in Fig. 12; the surrounding rock of the roadway essentially tended to be stable after 90 days. As shown in the figure, the roof subsidence amount of the sump was 17.2–19.2 mm; the floor heave amount was 13.9–16.5 mm; and the convergence between the two sides of the sump was 23.2–27.9 mm. There was no visible deformation on the surface of the sump, which fully met the use conditions.

After the completion of the drainage system in the floor rock strata under the 2127 goaf, the problems regarding the continuous water gushing in the follow-up working face and goaf of the No. 1 mining district at the − 980 mining level were effectively solved. When extensive water gushing occurred in the 2222 up-dip working face of the No. 2 mining district at the No. 2 mining level (the water inflow was up to 40,127 m^3^), the water would automatically flow into the drainage system, by excavating the 2222 drainage roadway. In addition, the drainage system was arranged under the goaf, which reduced the loss of mining resources and increased the economic benefits of the coal mining enterprise.

## Conclusions

Coal is an important energy source for developing China’s economy and industry. With the development trends in deep mining, safe and effective roadway maintenance with a considerable burial depth is of great significance for improving the economic benefits of coal mines. In this study, the adequate support of a sump buried at a depth of over 1200 m is taken as the research object, and the main conclusions are as follows.

1. The stress-release area formed in the floor rock strata of the working face 2127 after the working face 2127 was mined was a suitable location for arranging the permanent sump. This placed the sump in a stress environment with an average stress value of approximately 20 MPa. This realized overall pressure relief in the roadway-surrounding rock environment, with an overall pressure relief amplitude of 35.7%. Furthermore, the arrangement of the sump in this position significantly reduced the continuous deformation of the surrounding rock caused by the rheology of the deep rock mass and significantly reduced the difficulty of the surrounding rock control.
2. A panoramic drill hole camera system was used to observe the development of fissures in the temporary sump-surrounding rock under the supporting system, and pressure gauges were used to detect the anchor force of the bolts regularly. Combined with the failure characteristics of the surrounding rock on-site, the factors causing the instability of the temporary sump could be summarized as follows: ① a broken surrounding rock mass; ② the creep characteristics of the surrounding rock; ③ hydrological effects; and ④ support forms and parameters.
3. Based on the instability mechanisms of the temporary sump, a combined control technology was proposed, comprising the lengthened strong anchor bolts (cables), the full-section concrete-filled steel tubular support, and the pouring of full-section reinforced concrete and full-section long-hole grouting reinforcement. This technology could effectively improve the strength of the shallow broken surrounding rock and increase the support system's load-bearing capacity. From engineering practice, it was determined that there was no visible deformation on the surface of the sump after adopting the new support scheme. Thus, the large-deformation problem of the surrounding rock of the sump buried at a depth of over 1200 m was solved. In addition, this mature support technology provides theoretical and technical support for surrounding rock stability control in similar roadways.

## Data Availability

All data used to support the findings of this study are available from the corresponding author upon request.
